# Mitigating the SARS-CoV-2 Delta disease burden in Australia by non-pharmaceutical interventions and vaccinating children: a modelling analysis

**DOI:** 10.1186/s12916-022-02241-3

**Published:** 2022-02-18

**Authors:** George J. Milne, Julian Carrivick, David Whyatt

**Affiliations:** University of Western Australia, Crawley, WA Australia

**Keywords:** SARS-CoV-2, COVID-19, Mitigation strategy effectiveness, Modelling, Vaccination, Non-pharmaceutical measures, Revised 2nd January 2022

## Abstract

**Background:**

In countries with high COVID-19 vaccination rates the SARS-CoV-2 Delta variant resulted in rapidly increasing case numbers. This study evaluated the use of non-pharmaceutical interventions (NPIs) coupled with alternative vaccination strategies to determine feasible Delta mitigation strategies for Australia. We aimed to understand the potential effectiveness of high vaccine coverage levels together with NPI physical distancing activation and to establish the benefit of adding children and adolescents to the vaccination program. Border closure limited SARS-CoV-2 transmission in Australia; however, slow vaccination uptake resulted in Delta outbreaks in the two largest cities and may continue as international travel increases.

**Methods:**

An agent-based model was used to evaluate the potential reduction in the COVID-19 health burden resulting from alternative vaccination strategies. We assumed immunity was derived from vaccination with the BNT162b2 Pfizer BioNTech vaccine. Two age-specific vaccination strategies were evaluated, ages 5 and above, and 12 and above, and the health burden determined under alternative vaccine coverages, with/without activation of NPIs. Age-specific infections generated by the model, together with recent UK data, permitted reductions in the health burden to be quantified.

**Results:**

Cases, hospitalisations and deaths are shown to reduce by (i) increasing coverage to include children aged 5 to 11 years, (ii) activating moderate NPI measures and/or (iii) increasing coverage levels above 80%. At 80% coverage, vaccinating ages 12 and above without NPIs is predicted to result in 1095 additional hospitalisations per million population; adding ages 5 and above reduces this to 996 per million population. Activating moderate NPIs reduces hospitalisations to 611 for ages 12 and over, and 382 per million for ages 5 and above. Alternatively, increasing coverage to 90% for those aged 12 and above is estimated to reduce hospitalisations to 616. Combining all three measures is shown to reduce cases to 158, hospitalisations to 1 and deaths to zero, per million population.

**Conclusions:**

Delta variant outbreaks may be managed by vaccine coverage rates higher than 80% and activation of moderate NPI measures, preventing healthcare facilities from being overwhelmed. If 90% coverage cannot be achieved, including young children and adolescents in the vaccination program coupled with activation of moderate NPIs appears necessary to suppress future COVID-19 Delta-like transmission and prevent intensive care unit surge capacity from being exceeded.

**Supplementary Information:**

The online version contains supplementary material available at 10.1186/s12916-022-02241-3.

## Background

By May 2021 countries which had vaccinated significant proportions of their population, such as Israel, the UK and the USA, saw significant reductions in daily diagnosed SARS-CoV-2 case numbers [1]. From June 2021 onwards, the more transmissible B1.617.2 Delta variant became dominant worldwide, even in countries with high vaccination rates [2]. Given the rapid rise in Delta case numbers, the addition of children to COVID-19 vaccination schedules is under discussion by health authorities. Our model-based study aimed at understanding how expanding vaccination to younger age groups may reduce and contain this increase in case numbers, and consequential pressure on healthcare systems.

Mathematical modelling has been used successfully to inform vaccination policy, providing evidence on effectiveness of alternative vaccination strategies, such as the benefit of increasing influenza vaccination in children [3, 4]. We evaluated the similar role childhood vaccination may have in a COVID-19 Delta context and determined vaccine coverage levels needed to reduce and prevent growth in case numbers, with and without reintroduction of strict social distancing measures, as occurred in Australia in 2020 and 2021 [5].

## Methods

An individual-based model capturing the demographics and movement patterns of individuals within the Australian city of Newcastle (population ~273,000), together with SARS-CoV-2 virus transmission data from Wuhan, China, prior to social distancing activation [6], was previously developed. That model was used to analyse effectiveness of non-pharmaceutical social distancing interventions, varying their stringency, timing, and duration [7, 8]. The rationale for using a high-resolution agent/individual type model is its ability to determine the ages of those infected, with and without particular interventions activated. This permits estimation of inherently age-specific reductions in health burden outcomes, i.e. cases, hospitalisations, and deaths, resulting from activation of particular mitigation strategies.

### Transmission probability and reproduction number

Transmission between infectious and susceptible pairs of individuals in the model is stochastic, and a probabilistic transmission parameter governs the likelihood of an infectious individual transmitting the SARS-CoV-2 virus to a susceptible individual. The specific transmission parameter which produces a given population-wide basic reproduction number was obtained experimentally. This was achieved by first setting the probability of transmission between infectious and susceptible pairs of individuals to a specific probability, the pairwise transmission coefficient. Then a single random individual in the model was infected, with all other individuals in a susceptible state. The model software is then run and the number of secondary infections which occur are logged, and the process repeated multiple times to overcome stochastic uncertainty. The resulting secondary infection distribution provided the mean population-wide basic reproduction number resulting from a given transmission coefficient setting. This process was conducted repeatedly after adjusting the transmission parameter up or down, until the target basic reproduction *R*_0_ of 6.0 (95% CI [5.8, 6.2]) was obtained.

### Age-specific population demographics

Census data was used to capture the age-specific demographics of every individual in each household in the modelled community, assigning adults to workplaces, and children to age-specific classes [9–12]. Individual-based model technology captures the dynamics of the time-changing mobility and contact patterns of individuals, and the dynamics of virus transmission occurring between pairs of infectious and susceptible individuals. Transmission occurs when such pairs come into contact at two time points each day, to reflect occurrence of transmission in places of residence, i.e. households, on the one hand, and outside the home due to contact occurring in places of employment, schools, and in the wider community, on the other. Model development involved assigning individuals in one of 10 age classes to specific households in a given geographic area, to match Australian census data for the city of Newcastle. Children were allocated to age-specific classes in neighbouring schools, and adults to workplaces, again using census and other government datasets. Movement of individuals between these contact locales, and in the wider community, is modelled by explicitly moving their location out-with their household during a daytime phase and then having them return to their household in an evening phase.

### Health burden outcomes

The model captures the age of each modelled individual in one of 10 age bands, allowing age-specific health outcomes to be determined following infection [13]. Model outputs capture the infection history of all individuals in the community, representing where and when infections occur. The modelling analyses quantified infection and case reductions arising from increased vaccination coverage, and thus, the reduction in hospitalisations and deaths.

For the purposes of estimating hospitalisations and deaths, infection data generated by the model was translated into cases using a 2:1 ratio, a proportion chosen to reflect estimated infection/case ratios in Australia in 2021 [14]. Hospitalisations and deaths were derived from case data using United Kingdom (UK) Delta variant health burden datasets [15, 16]. Application of UK data was necessary given the much larger SARS-CoV-2 Delta infection rate in the UK compared to Australia and is appropriate given similar population demographics and health systems in Australia and the UK. We used the large UK COVID-19 health burden datasets from June 15, 2021, to August 27, 2021, when (effectively) all SARS-CoV-2 transmission was due to the Delta variant [15]. The derived age-specific case/hospitalisation and case/fatality ratios are given in Additional file 1: Table S1.

The agent-based model represents the ages of each of the 273,407 persons in the modelled community (Newcastle, Australia) in one of 10 age bands, collected in the Australian census and accessed from the Australian Bureau of Statistics [9, 10]. Age-specific UK COVID-19 Delta variant health burden data was used to establish age-specific case/hospitalisation and case/mortality ratios and then used to assign age-specific health burden outcomes to each of the individuals infected within the model. These ratios are detailed in Additional File 1: Table S1. All health burden outcomes were scaled up to a population size of 1 million, allowing results to be readily translated to population centres of different sizes.

### Vaccination

We modelled the BNT162b2 Pfizer vaccine with assumed protection against Delta infection of 88%. This percentage of individuals is assumed to remain immune for a period of at least 6 months, without immunity waning [17]. The Pfizer vaccine affords protection against symptomatic disease by the Delta variant lower than for the Wuhan strain [7] and the B.1.1.7 (Alpha) variant, reducing from 93% (Alpha) to 88% (Delta) [17]. In the absence of further evidence, we assumed protection against infection to be the same as protection against disease. In the model the effect of vaccination is captured at the individual level, where a certain percentage of fully vaccinated individuals “move” from a susceptible state to an immune state. The percentage of individuals moving to an immune state thus reflects vaccine efficacy. Further model parameters are presented in Table 1.

**Table 1:** UWA COVID-19 model parameter settings. Where applicable, the data source is referenced after the value. Estimated values in the absence of applicable studies are described in the text

Model population	273,407. 2011 census data for Newcastle and Lake Macquarie East, NSW.
**Assumed delta variant basic reproductive number**	6.0 (95% CI [5.8, 6.2]) [2]
**Time from infection to symptoms**	5 days [18]
**Time from infection to infectious**	3 days [18]
**Time from infection to recovery**	9 days (4 days of symptoms, 6 days being infectious) [18]
**Child (<12 years) susceptibility**	Same as adults and adolescents
**Child (<12 years) transmissibility**	50% of adults
**Adolescent (12–17 years) transmissibility**	Same as adults
**Probability of asymptomatic infections**	35% [19]
**Probability of asymptomatic transmission**	55% of symptomatic transmission [20]
**Probability of symptomatic infection isolation**	33%
**Home isolation of diagnosed cases**	7 days
**Seeded infections**	On average 1 infection every 4 days for 30 days
**BNT162b2 Pfizer vaccine**	Efficacy: 88% (against infection and transmission)
**Trigger for NPIs**	1 diagnosed case in a single day
**Moderate NPIs**	**School closures**: 0% **Workplace non-attendance**: 20% **Community contact reduction**: 60%
**Strict NPIs**	**School closures**: 100% **Workplace non-attendance**: 50% **Community contact reduction**: 80%
**Overall hospitalisation/ICU ratio**	5:1 [21]

The effectiveness of a particular vaccination strategy was determined by running model software after adjusting vaccination settings for alternative coverage levels and ages vaccinated. Seeding infectious individuals into the model initiated a COVID-19 Delta outbreak in a COVID-19 naive population that had been vaccinated to a given coverage level [8]. Thus, a Delta variant outbreak was assumed to occur after all vaccinations had been completed and was modelled by the short-term seeding of infectious individuals into randomly selected households. This introduction of infectious individuals into the modelled population occurred at the rate of a single infection every 4 days for 30 days, equivalent to 4 infections seeded in a population of 1 million every fourth day.

The study assumed an Australian setting with effectively no immunity resulting from infection, due to early international border closures and adoption of a SARS-CoV-2 elimination policy. We thus assume all immunity effects are attained as the result of vaccination, rather than immunity derived from infection. Australia adopted an age-specific vaccination strategy in March 2021 due to limited supplies of mRNA vaccines, with those aged 60 year and over vaccinated with the AstraZeneca vaccine, manufactured locally, with all younger age groups vaccinated with the Pfizer vaccine. Our study assumed a modified vaccination strategy by using the Pfizer mRNA vaccine to boost the immune response of those previously vaccinated with the AstraZeneca vaccine, necessary due to its lower efficacy against the Delta variant [17, 22–24]. This approach allowed us to simplify the modelled scenarios by assuming all vaccinated individuals had either a second or third dose of the Pfizer vaccine, and thus, the same protective effect following their most recent vaccination. From October 2021 Australia introduced the Pfizer vaccine to boost immunity levels for those vaccinated 6 months previously with either the Astra Zeneca or Pfizer vaccines.

We assumed that two doses of the Pfizer vaccine gave protection against Delta infection of 88%. Protection against poor health outcomes in those vaccinated and having breakthrough infections was implicitly included by using recent UK Delta variant data [15] to give age-specific case/hospitalisation and case/mortality ratios, as in Additional file 1: Table S1.

### Parameter settings

Model parameter settings listed in Table 1 were based on values from published studies where possible. Due to the discrete nature of the model parameters, where source data are presented as a probability distribution either the mode or a truncated range was used. In the absence of data relevant to the Delta variant, we have made the following assumptions. Child and adolescent susceptibility is taken to be the same as adults, based on the high levels of cases in these age groups observed in the UK [15]. Child transmissibility is assumed to be 50% that of adults due to the reduced level of symptoms observed in this age group [25]. The probability of a symptomatic case isolating is hard to estimate, varying greatly according to severity of symptoms, and testing and home isolation compliance. A value of 33% was chosen to reflect a high proportion of cases with mild symptoms and a moderate/low level of testing and isolation compliance. In practice, it was observed that adjusting this value had little effect on the results, most likely due to a large proportion of transmission occurring in the pre-symptomatic period [20]. Home isolation of diagnosed cases was set at 7 days to conservatively cover the infectious period following symptom onset. Further details of the model are presented in Additional file 1.

The median value of 100 simulations is presented in the following “Results” section. Multiple simulations are necessary given the inherent stochasticity of person-to-person virus transmission and in the infection seeding procedure. Confidence intervals were found to be narrow. To aid clarity, confidence intervals are not presented in the following tables and are provided alongside the results data in Additional file 1.

Results are presented for vaccination scenarios under alternative vaccine coverages, varied from 70 to 90% in individuals in two age categories: 5 and above, and ages 12 and above. We also consider activating moderate physical distancing, non-pharmaceutical (NPI) measures when Delta cases first appear in a community. These correspond to the Stage 3 measures adopted by the State of Victoria, Australia, in 2020 and 2021, estimated as 20% workplace non-attendance and 60% community contact reduction [8], altered to include schools remaining open. This is termed moderate NPIs in Table 1. Strict NPIs in Table 1 correspond to Victoria’s Stage 4 lockdown measures.

## Results

An overview of the results is presented in Table 2, allowing the effectiveness of alternative vaccination strategies to be compared, with and without concomitant non-pharmaceutical interventions (NPIs). These data suggest that increasing childhood vaccinations to include ages 5 to 11, in addition to adolescents aged 12 and above, gives a further substantial reduction in cases, hospitalisations and deaths. The rapid activation of strict physical distancing NPIs involving substantial economic, educational and societal disruption with schools closed (as defined in Table 1) is shown in Table 2 to substantially reduce the disease burden of highly transmissible COVID-19 variants such as Delta, *R*_0_=6.0. The use of moderate, less economically disruptive NPIs with schools remaining open, can be seen to be effective in reducing the health burden when compared to relying on vaccination alone, i.e. without activation of NPI measures. This is particularly evident at the highest vaccine coverage levels considered, 90%.

**Table 2:**
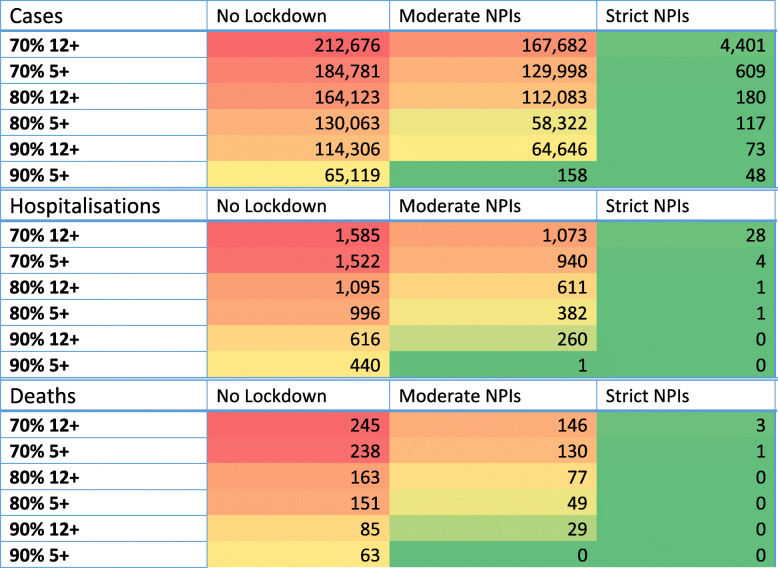
Total cases, hospitalisations and deaths per 1,000,000 population. Transmission calibrated to R0 of 6.0. Vaccine efficacy of 88% assumed for all ages. Median value of 100 simulations presented. Colours are linearly distributed according to the value along a minimum (green)/median (yellow)/maximum (red) scale

**Fig. 1:**
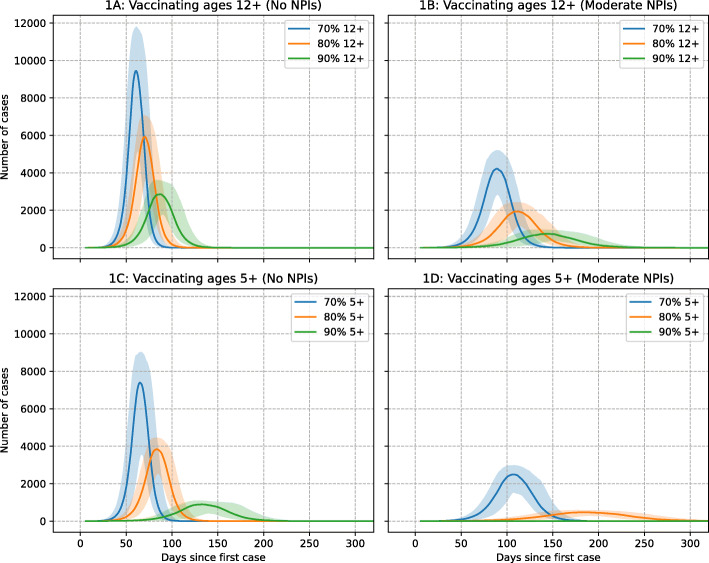
Daily cases per million for alternative vaccine coverage levels, with and without moderate NPIs. Vaccine with 88% efficacy assumed for all ages. *R*_0_ =6.0. Median value of 100 simulations presented, with 10*th* and 90*th* percentile as shaded areas

Predicted case data illustrated by epidemic curves in Fig. 1 indicate that altering vaccination policy to include ages 5 to 11, in addition to adolescents aged 12 and above, is significant in terms of reducing daily case number growth and consequential pressure on healthcare resources. Reduction in estimated hospitalisation numbers achieved by alternative mitigation strategies is presented in Table 4.

In the absence of physical distancing measures (Fig. 1A, C), a vaccination strategy which includes children aged 5 and above is estimated to reduce the peak in daily case numbers by approximately 2000 per 1 million population, for coverage levels of 70% and 80%. At 80% coverage, a peak of ~6000 cases per million with vaccination of years 12 and above is predicted to reduce to a maximum of ~4000 per million per day if vaccinating ages 5 and above are added to the vaccination program. At 90% coverage, the benefit of including younger children is more pronounced, reducing the peak in cases by over half, from ~3000 to ~1000 per million population. With the addition of moderate physical distancing measures, which involve schools remaining open, Fig. 1B, D further highlights the benefit of vaccinating these younger age groups.

Data in Table 2 show that at a coverage level of 80% in those aged 12 and above, the total number of cases in a population of 1 million is estimated to reduce from ~164,000 to ~130,000 without NPI measures activated, and from 112,000 to 58,000 with moderate lockdown activated, when vaccinating children aged 5 and over is included, per million population. These data illustrate the reduction in the COVID-19 health burden, i.e. of cases and hospitalisations, and thus, reduction in excess health system demand due to COVID-19. At 80% coverage, hospitalisations and deaths are predicted to reduce slightly without moderate physical distancing measures. With moderate measures added, hospitalisations are seen to reduce from 611 to 382 per million, while deaths are predicted to reduce from 77 to 49 per million population. Data in Additional file 1: Tables S2, S3, and S4 present the percentage reduction in cases, hospitalisations and deaths estimated to occur when ages 5 and 11 years are added to the vaccination program, when moderate NPIs are activated, and when coverage levels are higher than 70%.

As seen in Table 2, at 80% vaccine coverage of ages 12 and above without moderate NPIs, two strategies may be adopted to reduce the health burden further: activating moderate NPIs or increasing vaccine coverage. Activating moderate physical distancing measures is shown to reduce hospitalisations to 611 per million. Alternatively, increasing coverage to 90% results in a reduction to 616 hospitalisations per million. Thus, activating moderate NPIs is seen to give a similar reduction in hospitalisations as increasing coverage of ages 12 and above to 90%.

Tables 3, 4 and 5 present the reduction in the COVID-19 health burden by age group bands. Cases, hospitalisations and deaths are estimated to reduce by (i) increasing vaccine coverage to include children aged 5 to 11 years, (ii) activating moderate physical distancing measures and/or (iii) increasing coverage levels above 80%. To reduce pressure on health systems, hospitalisation rates may be reduced by combining all or some of these 3 strategies. Tables S2, S3 and S4 in Additional file 1 present the percentage reduction in health burden metrics achieved by the above strategies. Tables S5, S6 and S7 in Additional file 1 present the same data with associated confidence intervals.

**Table 3:** Cases per million for increasing vaccination coverage levels (ages 12 and up, and 5 and up), with and without moderate NPIs. Vaccine with 88% efficacy assumed for all ages. *R*_0_ =6.0. Median value of 100 simulations presented

Cases		Total	0–12 years	13–24 years	25–44 years	45–64 years	65–79 years	80y+
No NPIs	70% 12+	212,676	67,404	29,831	46,467	43,853	17,480	7589
	70% 5+	184,781	42,533	29,487	45,412	42,966	17,026	7372
	80% 12+	164,123	63,021	21,026	33,165	30,391	11,514	4985
	80% 5+	130,063	34,751	20,252	31,058	28,707	10,694	4583
	90% 12+	114,306	57,750	11,773	19,413	16,791	5962	2547
	90% 5+	65,119	21,972	9401	14,585	12,881	4381	1869
Moderate NPIs	70% 12+	167,682	58,849	24,250	37,429	32,826	10,116	4107
	70% 5+	129,998	31,373	22,789	33,274	29,880	8992	3632
	80% 12+	112,083	49,915	13,918	22,350	18,474	5233	2114
	80% 5+	58,322	16,791	9973	14,465	12,422	3356	1319
	90% 12+	64,646	39,580	5361	9813	7200	1906	759
	90% 5+	158	50	18	35	28	8	2

**Table 4:** Hospitalisations per million for increasing vaccination coverage levels (ages 12 and up, and 5 and up), with and without moderate NPIs. Vaccine with 88% efficacy assumed for all ages. *R*_0_ of=6.0. Median value of 100 simulations presented

Hospitalisations		Total	0–12 years	13–24 years	25–44 years	45–64 years	65–79 years	80 years+
No NPIs	70% 12+	1585	65	46	298	426	355	394
	70% 5+	1522	39	46	291	417	346	383
	80% 12+	1095	62	33	213	295	234	259
	80% 5+	996	32	31	199	279	217	238
	90% 12+	616	57	18	125	163	121	132
	90% 5+	440	20	15	94	125	89	97
Moderate NPIs	70% 12+	1073	58	38	240	319	206	213
	70% 5+	940	30	35	213	290	183	189
	80% 12+	611	50	22	143	179	106	110
	80% 5+	382	17	15	93	121	68	68
	90% 12+	260	41	8	63	70	39	39
	90% 5+	1	0	0	0	0	0	0

**Table 5:** Deaths per million for increasing vaccination coverage levels (ages 12 and up, and 5 and up), with and without moderate NPIs. Vaccine with 88% efficacy assumed for all ages. *R*_0_ of=6.0. Median value of 100 simulations presented

Deaths		Total	0–12 years	13–24 years	25–44 years	45–64 years	65–79 years	80 years+
No NPIs	70% 12+	245	0	1	4	42	76	121
	70% 5+	238	0	1	4	42	74	117
	80% 12+	163	0	1	3	29	50	79
	80% 5+	151	0	1	3	28	47	73
	90% 12+	85	0	0	2	16	26	40
	90% 5+	63	0	0	1	12	19	30
Moderate NPIs	70% 12+	146	0	1	4	32	44	65
	70% 5+	130	0	1	3	29	39	58
	80% 12+	77	0	0	2	18	23	34
	80% 5+	49	0	0	1	12	15	21
	90% 12+	29	0	0	1	7	8	12
	90% 5+	0	0	0	0	0	0	0

At 80% coverage, vaccinating ages 12 plus without NPIs is predicted to result in 1095 hospitalisations per million population, adding ages 5 plus reduces this to 996 per million, see Table 4. If moderate NPIs are applied at 80% coverage of ages 5 and above, case numbers are predicted to reduce by ~50 to 58,322; hospitalisations to reduce by ~50 to 382; and deaths reduce from 151 to 49, all per million population. Combining all three measures; *viz*. vaccination children aged 5 to 11, activating moderate NPIs and reaching 90% coverage, is shown in Tables 2, 3, 4 and 5 to reduce total cases to 158, hospitalisations to 1 and deaths to zero, per million of the population.

### Healthcare demand

A key aim of COVID-19 mitigation is to lessen the mortality rate, and the demand on the healthcare system. With hospital bed resources occupied by COVID-19 patients there are significant effects more generally, negatively impacting access to healthcare for medical conditions other than those associated with COVID-19.

**Fig. 2:**
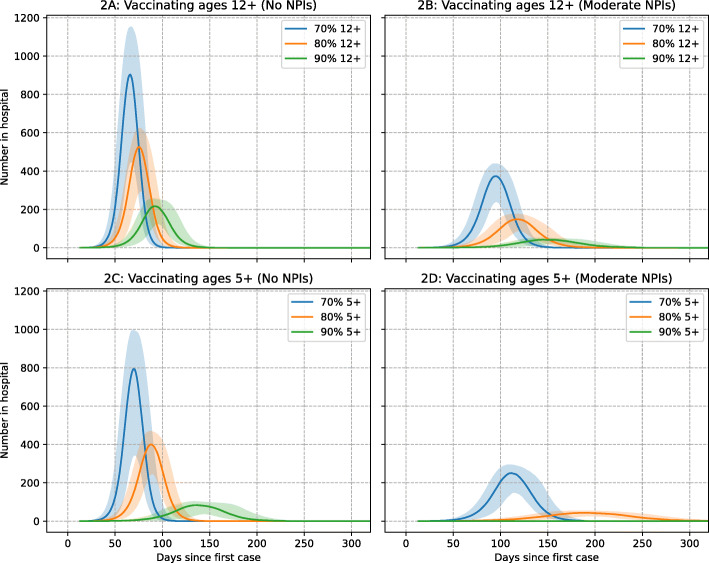
Two-week sum of hospitalisations per million population illustrating peak hospital demand for alternative vaccine coverage levels, with and without moderate NPIs. Median value of 100 simulations presented with 10th and 90th percentile shaded. Hospitalisations include admissions to general wards, high-dependency units and ICUs

Excess hospital demand, as illustrated in Fig. 2, was estimated using a rolling 2-week sum of daily hospitalisations, with average duration taken from 2021 UK Delta variant data sources [26]. Peak intensive care unit (ICU) bed demand was determined using Australian hospitalisation and ICU data from September and October 2021, when all COVID-19 hospitalisations were due to the Delta variant, giving a 5:1 ratio of total COVID-19 hospitalisations to ICU bed occupancy [21].

From Fig. 2, and in the absence of further interventions, 80% vaccination coverage of ages 12 and above are estimated to result in daily peak hospital bed demand of ~500 beds per million, of which ~100 are ICU beds. Adding moderate NPI measures is estimated to reduce hospital demand to ~150 beds per million, of which ~30 relate to ICU admissions. With the addition of ages 5–11 to the vaccination program, and 80% coverage, we estimate ~400 beds per million without NPIs, of which ~80 are ICU beds. This reduces to ~50 beds per million with moderate NPI measures added, of which ~10 are required in ICU.

At 90% vaccine coverage of ages 12 and above, and without activation of moderate NPIs, hospital demand is estimated to peak at ~200 per million, of which ~40 would be in ICU. Vaccinating ages 5 to 11 years in addition reduces peak hospitalisations by half, to ~100 of which ~20 would be admitted to ICU. If moderate NPIs are applied, peak hospitalisation demand at 90% coverage of ages 12 and above is estimated at ~50 per million, with ~12 of these in ICU. If ages 5 to 11 are also vaccinated, Fig. 2D indicates that excess hospital demand approaches zero.

### ICU demand

The ability of countries to manage surges in cases due to highly transmissible variants is highly dependent on intensive care unit (ICU) capacity, rather than general ward capacity. A survey conducted by the Australian and New Zealand Intensive Care Society from 30th August to September 9, 2021, provides data on additional ICU bed and staff resources in Australian ICUs which will permit capacity to increase in response to increased pandemic demand [27]. Using ICU data from this survey, we further analysed the response scenarios detailed in Fig. 2 to determine which mitigation strategies prevent ICU facilities from being overwhelmed following a surge in COVID-19 cases.

The following ICU survey data are pertinent: Australia’s maximum ICU bed capacity of 5625 includes 2183 currently staffed beds, 813 additional physical ICU beds, and 2627 in surge areas outside ICUs; however, available nursing staff would only permit 383 additional ICU beds to open at pre-pandemic staffing levels [27]. Scaling the previous estimates of ICU bed demand at both 80% and 90% vaccine coverage levels to the total Australian population (~25.75 million), we determined the mitigation strategies which held excess ICU bed demand below the 383 fully staffed bed threshold.

At 80% vaccination coverage the only effective mitigation strategy to keep ICU demand below this threshold was to vaccinate ages 5 and above and to activate moderate NPIs, requiring ~257 additional ICU beds. Vaccination at 80% coverage without the addition of moderate NPI measures was found to significantly exceed excess ICU capacity, requiring ~2575 additional beds when vaccinating ages 12 and above, and ~2060 when vaccinating ages 5 and above.

At 90% vaccination coverage, vaccinating both age groups with moderate NPIs activated is estimated to keep excess ICU demand below the threshold, requiring ~309 additional ICU beds when vaccinating ages 12 and above, and no additional ICU beds if vaccinating ages 5 and above. In the absence of moderate NPIs, and at 90% coverage, vaccinating ages 12 and above is estimated to require ~1030 additional ICU beds, and ~515 if, in addition, ages 5 to 11 years are also vaccinated; both vaccination strategies exceed the 383 available fully staffed

Staff resources may permit ICU facilities to exceed the 383 ICU bed threshold and open all 813 additional available ICU beds to meet excess demand, at least as a short-term response. With this increased availability of ICU beds, vaccinating both age groups at *80% coverage and above* together with activation of moderate NPIs, keeps ICU requirements below this extended 813 ICU bed threshold. If moderate NPIs are not activated, vaccinating 80% of both age groups increases case and hospitalisation numbers such that ICU demand significantly exceeds the extended ICU capacity; requiring ~2575 beds if vaccinating ages 12 and above, and 2060 if vaccinating ages 5 and above. At 90% coverage, and in the absence of moderate NPIs, vaccinating ages 5 and above is estimated to reduce excess ICU bed demand to ~515 beds, below the 813 extended bed threshold. If vaccinating 90% of ages 12 and above without attendant moderate NPIs activated, excess ICU bed demand is estimated at ~1030 and thus exceeds the extended threshold.

## Discussion

In the context of a highly transmissible SARS-CoV-2 variant, this study conducted a comprehensive modelling analysis of the population-wide effectiveness of a range of alternative vaccination strategies. These involved vaccination-only strategies, and those with attendant physical distancing non-pharmaceutical measures, under a range of vaccine coverage levels. The aim was to determine vaccination strategies which best mitigate the spread of a highly transmissible SARS-CoV-2 variant (i.e. B1.617.2 Delta), reduce the resulting COVID-19 health burden, and lessen pressure on healthcare systems.

The use of an agent-based model (*c.f*. individual-based model) permitted us to calculate the age-specific health burden prevented under a wide range of mitigation settings. The ages of those whose infections were prevented under a given mitigation strategy was used to determine the reduction in the inherently age-specific hospitalisation and mortality numbers, when coupled with known case/hospitalisation and case/fatality data.

### Health policy implications

Results from our study reinforce the message that at a basic reproduction number of approximately 6.0, vaccination alone cannot control such highly transmissible variants. Hence non-pharmaceutical measures are also required to reduce transmission by reducing direct person-to-person contact. This is in contrast to the situation with the COVID-19 Alpha variant (*R*_0_ = 2.9) where significant indirect protection can be observed at 80% vaccination coverage of ages 5 and above with no NPIs, and 70% coverage of ages 12 years and above if moderate NPIs are activated, see Additional file 1: Table S9. Similarly, for an early estimate of Delta variant transmissibility of *R*_0_ = 4.0, 90% vaccination coverage of ages 5 years and above without activation of NPIs, or 80% coverage with moderate NPIs activated, are shown to result in significant indirect protection, see Additional file 1: Table S10.

Key findings of the study suggest that significant outbreaks of SARS-CoV-2 Delta-type variants may continue to occur unless vaccination reaches very high coverage levels in adults, adolescents and school-age children. Our study further suggests activation of physical distancing NPI measures will still be required, even in populations with high vaccination levels, and activating such measures will be required as soon as case numbers are seen to increase. The addition of children aged 5 to 11 to the vaccination program was also found to be beneficial. Combining all three measures; *viz*. vaccination children aged 5 to 11, activating moderate NPIs, and reaching 90% coverage, is shown to effectively halt transmission of a Delta-like variant.

Results provide evidence that moderate lockdown measures with schools remaining open may successfully contain future high transmission variants under achievable coverage levels. They further highlight the need to activate such measures as early as possible. We determined that vaccinating adolescents and younger children was critical, to both increase the pool of immune individuals, and to mitigate transmission in an age group implicated in high transmission of viruses by aerosol droplets, as is the case with influenza [4]. While strict, hard lockdown measures are shown to arrest SARS-CoV-2 spread, they are known to cause significant societal and economic disruption, and negatively impact on children’s education. However, moderate physical distancing measures with schools remaining open, as presented here, should be accepted by the public in contrast to more severe measures, and acceptable by health authorities given their role in mitigating virus transmission.

### Reducing the health burden

At 80% vaccine coverage of ages 12 and above, activating moderate NPIs is estimated to reduce hospitalisations to a similar level as may be achieved by increasing coverage of ages 12 and above to 90%. If this level of coverage proves hard to achieve, then use of short-term moderate NPI measures may be applied as a response to growing case numbers. Conversely, if vaccination rates are at 90% or over, activation of NPIs may not be required if the resulting excess hospitalisations are manageable within a particular health system. From an economics perspective, the cost of even moderate physical distancing measures may be significantly larger than increasing vaccination rates by an additional 10% due to the shutdown of hospitality and other workplaces. Future research should aim to better understand such economic trade-offs.

The study quantified COVID-19 vaccination targets that may substantially reduce the growth in case numbers and resulting health burden, and which avoid the need for damaging hard lockdown measures. Adding adolescents to the vaccination schedule was found to be crucial in achieving these targets, while the addition of children ages 5 to 11 was also found to be highly effective in reducing the COVID-19 health burden. Vaccinating children and adolescents has key benefits; it increases the overall population-wide coverage level, and furthermore, it reduces transmission in an age group implicated in high transmission of viruses by aerosol droplets in school settings, e.g. influenza [4]. Allowing Delta outbreaks to be successfully managed via a combination of achievable coverage rates and moderate physical distancing measures, those that allow schools and many workplaces to remain open, will minimise repeated and enduring hard lockdown measures and resulting economic, health and educational damage. Further research is required to establish the costs and benefits of such COVID-19 mitigation policies.

### COVID-19 hospital demand

In the absence of physical distancing NPI measures, we predict that even with a high 90% vaccine coverage in those aged 12 and above, significant outbreaks of over ~550,000 cases in a population of 5 million is possible, cities the size of Sydney and Melbourne, Australia, see Additional file 1: Table S8. Furthermore, our modelling suggests that ~3000 hospitalisations and over 400 deaths may result in these large cities. At a lower 80% vaccination coverage, over 820,000 cases are estimated in the absence of any physical distancing measures, approximately 16% of the population. At its peak we estimate ~30,000 new cases per day (extrapolating from Fig. 1) which are estimated to result in a peak demand of ~2500 hospital beds, of which ~500 may be required in ICU facilities, by extrapolation from Fig. 2.

The addition of moderate NPIs coupled with 80% vaccine coverage in ages 12 and above was found to reduce cases to ~560,000 in a population of 5 million, ~11% of the population. This level of vaccination would see peak hospital bed demand at ~750 (from Fig. 2) with a total of 385 deaths estimated, see Additional file 1: Table S8. These estimated case, hospitalisation and death numbers are similar to the reduction which may be achieved at 90% vaccine coverage in ages 12 and above without NPI measures. These data suggest that attempts to mitigate outbreaks of highly transmissible COVID-19 variants without attendant social distancing measures will result in significant numbers becoming infected, unless vaccine coverage significantly greater than 80% can be achieved. The inclusion of children aged 5 to 11 years is shown to reduce the COVID-19 health burden, by increasing the “pool” of immune individuals in the overall population and reducing transmission in school settings. Vaccinating school age children and adolescents has the added advantage that vaccination may be conducted efficiently within schools, as occurs for other infectious diseases in middle- and high-income countries.

From the perspective of managing the daily peak in hospitalised COVID-19 cases, the study estimates that moderate NPI measures may reduce COVID-19 hospital demand from ~500 to ~150 at 80% coverage of ages 12 and above, and from ~200 to ~50 at 90% coverage, all per million population. Of these hospitalisations, application of moderate NPIs is estimated to reduce ICU bed demand from ~100 beds to ~30 per million at 80% coverage, and at 90% coverage giving a reduction from ~40 ICU beds without NPIs, to ~10 when moderate NPIs are activated, all per million population. With the addition of ages 5–11 to the vaccination program and at 90% coverage, we estimate ~20 ICU beds without NPIs, reducing to near zero ICU beds with moderate NPI measures added, all per million population.

A 2021 survey of all 193 ICU facilities in Australia indicates that 2183 staffed ICU beds are currently available, with 813 additional surge capacity beds available within ICU facilities [27]. As highlighted in that study, availability of additional critical care staff resources restricts this additional surge capacity to 383 staffed ICU beds. The ability of Australia (population ~25.75 million) to provide adequate patient care in ICU facilities during future Delta-like surges will thus be dependent on high vaccination rates and activation of NPIs. Given this staffed ICU bed availability, mitigation measures are needed to keep excess ICU demand to less than 16 per million via activation of moderate NPI measures and/or the inclusion of children aged 5 to 11 years in the vaccination program. If additional critical care staff were available, a maximum of ~32 fully staffed ICU beds may be provided for severe COVID-19 cases, per million population. A minimum vaccination coverage of over 90% of ages 12 and above would be required to stay within this limit. However, vaccinating children aged 5 to 11 years or activating moderate NPIs would reduce pressure on this vaccination threshold.

At 80% vaccination coverage the only effective mitigation strategy found to keep ICU demand below the current fully staffed additional ICU bed resource of 383 beds Australia-wide was to vaccinate ages 5 and above and to activate moderate NPIs. At 90% vaccination coverage, vaccinating both age groups with moderate NPIs activated is estimated to keep excess ICU demand below the currently available bed threshold.

If staffing resources were increased to allow use of all 813 additional physical ICU beds at pre-pandemic staffing levels this effectively doubles available ICU surge capacity. In this situation, vaccinating either age group at 80% coverage (or more) when coupled with activation of moderate NPIs is estimated to keep ICU requirements below this extended ICU bed threshold. At 90% coverage and without moderate NPIs activated, vaccinating children aged 5 and to 11 in addition to those aged 12 and above is found to be necessary to reduce excess ICU bed below the 813 extended bed threshold.

These findings reinforce the importance of activating non-pharmaceutical social distancing intervention measures as a means of preventing critical care hospital facilities from being overwhelmed during a surge in COVID-19 cases, even with high vaccination coverage levels.

Adoption of moderate NPIs is estimated to give a reduction of 40–70% of cases, hospitalisations and deaths in the elderly for all vaccination coverage levels evaluated. An exception is at 90% coverage of ages 5 and over, where data suggests that transmission is effectively eliminated. This older age cohort are at higher risk of poor health outcomes following infection compared to other age groups [28], thus use of even moderate NPIs should permit a significant reduction in their hospitalisations and deaths.

Results provide evidence that moderate physical distancing measures, which importantly allow schools to remain open, can successfully contain future high transmission variants under achievable vaccination coverage levels above 80%. They further highlight the need to activate such NPI measures as early as possible, as found in a prior study [8]. Results are consistent with a study which found that vaccine-only strategies were unlikely to achieve herd-immunity in Australia, a study that evaluated vaccination in the context of the less transmissible Alpha variant, with an *R*_0_ of 2.9 [29]. A further study by the same authors evaluated the current Delta outbreak in Sydney, Australia, using a basic reproduction number of 5.97 [30]. That study evaluated the progressive increase in vaccine coverage rates and also suggests the need for concomitant NPI measures to be applied.

Findings from our study are based on a number of assumptions, including the assumed transmission rate of the Delta variant, vaccine effectiveness against infection, and the rapid response of activating physical distancing measures once case numbers are first seen to increase. We have chosen to assume ongoing use of booster vaccinations and thus minimise the effect of waning vaccine immunity. This simplified our analyses and allows the benefit of higher vaccination coverage and attendant NPIs to be directly compared, particularly given the limited data available for vaccine waning characteristics, and whether they would be age specific.

Results from our model-based analyses may be scaled to reflect populations in high-income countries with demographics, economies and healthcare systems similar to Australia. These would include many European countries, the USA, Canada, and parts of the Asia Pacific region.

### Future studies

Future research is required to analyse the impact of waning immunity, both from vaccination and infection, and the timing of a regular booster vaccination regimen. Age-specific estimates of health burden reduction arising from alternative mitigation strategies, such as those presented, may be applied in an economic analysis to determine the relative cost-effectiveness of these alternatives. Thus, future research will compare the costs and benefits of alternative vaccination strategies and application of attendant non-pharmaceutical interventions. A further avenue of research would be to evaluate differentially targeting additional SARS-C0V-2 vaccines, particularly in settings with limited vaccine supplies. For example, to determine thresholds of increased vaccination in children that lead to substantial indirect protection in the elderly, those who have a poorer immune response to vaccination, a feature which has become apparent in this study.

## Conclusions

This model-based study determined the effectiveness of a range of vaccination strategies aimed at mitigating the impact of highly transmissible SARS-CoV-2 variants. Given the lack of indirect “herd immunity” protection with the Delta variant, analyses indicate that very high vaccination rates are required to directly protect individuals. Vaccination of school-age children and adolescents is found to directly lessen the age-specific health burden resulting from ongoing transmission of Delta-like variants, with this inclusion helping protect the vulnerable elderly age cohort. While vaccinating adolescents directly increases the “pool” of immune individuals in the population, adding children aged 5 years to 11 years to the vaccination schedule is also effective in reducing the health burden associated with COVID-19. Vaccinating this younger age group was found to indirectly protect the more vulnerable age groups. At 90% vaccination coverage and greater, including vaccination of children was estimated to reduce hospitalisations and deaths in those aged 65 years and older by approximately 25%. The activation of moderate non-pharmaceutical interventions which allows schools to remain open is shown to further strengthen the effectiveness of high vaccination rates, further reducing virus transmission and consequential hospitalisations and deaths.

## Supplementary Information

**Additional file 1.** Tables and text in PDF format.

## Data Availability

All empirical data used in this study are publicly available and have been cited in the article. The simulation code is available online.

## References

[1] Coronavirus Resource Center. https://coronavirus.jhu.edu/map.html (Accessed 13 Jul 2021).

[2] Campbell F, Archer B, Laurenson-Schafer H, Jinnai Y, Konings F, Batra N, Pavlin B, Vandemaele K, van Kerkhove MD, Jombart T, Morgan O, le Polain de Waroux O (2021). Increased transmissibility and global spread of SARS-CoV-2 variants of concern as at June 2021. Eurosurveillance.

[3] Milne GJ, Kelso JK, Kelly HA, Huband ST, McVernon J (2008). A small community model for the transmission of infectious diseases: comparison of school closure as an intervention in individual-based models of an influenza pandemic. PLoS ONE.

[4] Milne GJ, Halder N, Kelso JK, Barr IG, Moyes J, Kahn K, Twine R, Cohen C (2016). Trivalent and quadrivalent influenza vaccination effectiveness in Australia and South Africa: results from a modelling study. Influenza Other Respir Viruses.

[5] Victorian coronavirus (COVID-19) data. https://www.dhhs.vic.gov.au/victorian-coronavirus-covid-19-data (Accessed 13 Jul 2021).

[6] Li Q, Guan X, Wu P, Wang X, Zhou L, Tong Y, Ren R, Leung KSM, Lau EHY, Wong JY, Xing X, Xiang N, Wu Y, Li C, Chen Q, Li D, Liu T, Zhao J, Liu M, Tu W, Chen C, Jin L, Yang R, Wang Q, Zhou S, Wang R, Liu H, Luo Y, Liu Y, Shao G, Li H, Tao Z, Yang Y, Deng Z, Liu B, Ma Z, Zhang Y, Shi G, Lam TTY, Wu JT, Gao GF, Cowling BJ, Yang B, Leung GM, Feng Z (2020). Early transmission dynamics in Wuhan, China, of novel coronavirus–infected pneumonia. New England J Med.

[7] Milne GJ, Xie S, Poklepovich D. A modelling analysis of strategies for relaxing COVID-19 social distancing. medRxiv. 2020:20107425.

[8] Milne GJ, Xie S, Poklepovich D, O’Halloran D, Yap M, Whyatt D (2021). A modelling analysis of the effectiveness of second wave COVID-19 response strategies in Australia. Sci Rep.

[9] Lake Macquarie - East. 2013. https://quickstats.censusdata.abs.gov.au/census_services/getproduct/census/2011/quickstat/11101 (Accessed 26 Nov 2019).

[10] Newcastle. 2013. https://quickstats.censusdata.abs.gov.au/census_services/getproduct/census/2011/quickstat/11103 (Accessed 26 Nov 2019).

[11] Counts of Australian Businesses, including Entries and Exits, June 2012 to June 2016. https://www.abs.gov.au/AUSSTATS/abs@.nsf/Lookup/8165.0Main+Features1Jun%202012%20to%20Jun%202016?OpenDocument= (Accessed 17 May 2018).

[12] NSW government school enrolments by head count (2004-2018). 2019. https://data.cese.nsw.gov.au/data/dataset/nsw-government-school-enrolments-by-head-count/resource/da0fd2ec-6024-3206-98d4-81a2c663664b (Accessed 17 May 2018).

[13] Kelso JK, Halder N, Milne GJ (2013). Vaccination strategies for future influenza pandemics: a severity-based cost effectiveness analysis. BMC Inf Dis.

[14] Phipps SJ, Grafton RQ, Kompas T (2020). Robust estimates of the true (population) infection rate for COVID-19: a backcasting approach. R Soc Open Sci.

[15] Coronavirus (COVID-19) latest insights: comparisons. 2021. https://www.ons.gov.uk/peoplepopulationandcommunity/healthandsocialcare/conditionsanddiseases/articles/coronaviruscovid19latestinsights/Overview (Accessed 31 Aug 2021).

[16] Estimates of the population for the UK, England and Wales, Scotland and Northern Ireland. 2021. https://www.ons.gov.uk/peoplepopulationandcommunity/populationandmigration/populationestimates/datasets/populationestimatesforukenglandandwalesscotlandandnorthernireland (Accessed 31 Aug 2021).

[17] Lopez Bernal J, Andrews N, Gower C, Gallagher E, Simmons R, Thelwall S, Stowe J, Tessier E, Groves N, Dabrera G, Myers R, Campbell CNJ, Amirthalingam G, Edmunds M, Zambon M, Brown KE, Hopkins S, Chand M, Ramsay M (2021). Effectiveness of COVID-19 vaccines against the B.1.617.2 (Delta) Variant. New England J Med.

[18] Kang M, Xin H, Yuan J, et al. Transmission dynamics and epidemiological characteristics of Delta variant infections in China. medRxiv. 2021:21261991.

[19] Sah P, Fitzpatrick MC, Zimmer CF, Abdollahi E, Juden-Kelly L, Moghadas SM, Singer BH, Galvani AP (2021). Asymptomatic SARS-CoV-2 infection: a systematic review and meta-analysis. Proc Natl Acad Sci.

[20] Wu P, Liu F, Chang Z, Lin Y, Ren M, Zheng C, Li Y, Peng Z, Qin Y, Yu J, Geng M, Yang X, Zhao H, Li Z, Zhou S, Ran L, Cowling BJ, Lai S, Chen Q, Wang L, Tsang TK, Li Z (2021). Assessing asymptomatic, presymptomatic, and symptomatic transmission risk of severe acute respiratory syndrome coronavirus 2. Clin Infect Dis.

[21] Severity of COVID-19 cases: hospitalisations, ICU and ventilators. 2021. https://www.covid19data.com.au/hospitalisations-icu (Accessed 29 Nov 2021).

[22] Borobia AM, Carcas AJ, Pérez-Olmeda M, Castaño L, Bertran MJ, García-Pérez J, Campins M, Portolés A, González-Pérez M, García Morales MT, Arana-Arri E, Aldea M, Díez-Fuertes F, Fuentes I, Ascaso A, Lora D, Imaz-Ayo N, Barón-Mira LE, Agustí A, Pérez-Ingidua C, Gómez de la Cámara A, Arribas JR, Ochando J, Alcamí J, Belda-Iniesta C, Frías J, Martínez de Soto L, Rodríguez Mariblanca A, Díaz García L, Ramírez García E, Seco Meseguer E, Stewart Balbás SM, Marín Candón A, García García I, Urroz Elizalde M, Monserrat Villatoro J, de la Rosa P, Sanz García M, López Crespo C, Mauleón Martínez V, de Madariaga Castell R, Vitón Vara L, García Rodríguez J, Buño A, López Granados E, Cámara C, Rey Cuevas E, Ayllon García P, Jiménez González M, Hernández Rubio V, Moraga Alapont P, Sánchez A, Prieto R, Llorente Gómez S, Miragall Roig C, Aparicio Marlasca M, de la Calle F, Arsuaga M, Duque B, Meijide S, García de Vicuña A, Santorcuato A, Expósito I, de Benito S, Andia J, Castillo C, Irurzun E, Camino J, Temprano M, Goikoetxea J, Bustinza A, Larrea M, Gallego M, García-Vázquez D, de la Hoz AB, Pérez-Nanclares G, Pérez-Guzmán E, Idoyaga E, Lamela A, Oteo J, Castillo de la Osa M, Hernández Gutiérrez L, Andrés Galván ME, Calonge E, Andrés Galván ME, Bermejo M, de la Torre-Tarazona EH, Cascajero A, Fedele G, Perea C, Cervera I, Bodega-Mayor I, Montes-Casado M, Portolés P, Baranda J, Granés L, Lazaar S, Herranz S, Mellado ME, Tortajada M, Malet M, Quesada S, Vilella A, Llupià A, Olivé V, Trilla A, Gómez B, González E, Romero S, Gámez FJ, Casals C, Burunat L, Castelló JJ, Fernández P, Bedini JL, Vila J, Aguilar C, Altadill C, Armadans L, Borras-Bermejo B, Calonge J, Camacho L, Feliu A, Gili G, Llorente C, Martínez-Gómez X, Otero-Romero S, Palacio E, Parés O, Pinós L, Plaza A, Riera-Arnau J, Rodrigo-Pendás JA, Sans C, Santos J, Torres G, Torrens M, Uriona S, Ballarin Alins E, Pérez Esquirol E, Vendrell Bosch L, Laredo Velasco L, Uribe López D, González Rojano E, Sánchez-Craviotto M, Rivas Paterna AB, Hernán-Gómez TI, Rodríguez Galán N, Gil Marín JA, Álvarez-Morales V, Navalpotro AB, Jiménez-Santamaría MD, Cardós MC, Hermoso E, García-Arenillas M, Pérez Macías N, Domingo Fernández A, López Picado A, Quiñones JM, Deidda N, García-Franco A, Torvisco JM (2021). Immunogenicity and reactogenicity of BNT162b2 booster in ChAdOx1-S-primed participants (CombiVacS): a multicentre, open-label, randomised, controlled, phase 2 trial. Lancet.

[23] Liu X, Shaw RH, Stuart AS (2021). Safety and immunogenicity report from the Com-COV Study – a single-blind randomised non-inferiority trial comparing heterologous and homologous prime-boost schedules with an adenoviral vectored and mRNA COVID-19 vaccine. Preprints with Lancet.

[24] Schmidt T, Klemis V, Schub D, et al. Immunogenicity and reactogenicity of a heterologous COVID-19 prime-boost vaccination compared with homologous vaccine regimens. medRxiv. 2021:21258859.

[25] COVID-19 Delta variant in schools and early childhood education and care services in NSW: 16 June to 31 July 2021 National Centre for Immunisation Research and Surveillance, NSW Government. https://www.ncirs.org.au/sites/default/files/2021-09/NCIRS%20NSW%20Schools%20COVID_Summary_8%20September%2021_Final.pdf.

[26] Faes C, Abrams S, Van Beckhoven D (2020). Time between symptom onset, hospitalisation and recovery or death: statistical analysis of belgian COVID-19 patients. Int J Env Res Public Health.

[27] Litton E, Huckson S, Chavan S, Bucci T, Holley A, Everest E, Kelly S, McGloughlin S, Millar J, Nguyen N, Nicholls M, Secombe P, Pilcher D (2021). Increasing ICU capacity to accommodate higher demand during the COVID-19 pandemic. Med J Australia.

[28] COVID-19 deaths by age group and sex. 2021. https://www.health.gov.au/resources/covid-19-deaths-by-age-group-and-sex (Accessed 27 Jul 2021).

[29] Zachreson C, Chang SL, Cliff OM, Prokopenko M (2021). How will mass-vaccination change COVID-19 lockdown requirements in Australia?.

[30] Chang SL, Cliff OM, Prokopenko M (2021). Nowcasting transmission and suppression of the Delta variant of SARS-CoV-2 in Australia.

