# Supplementary material for: Mitigating the SARS-CoV-2 Delta disease burden in Australia by non-pharmaceutical interventions and vaccinating children: a modelling analysis
Source: BMC Med. 2022 Feb 18;20:80. doi: 10.1186/s12916-022-02241-3 (PMC8853841; doi:10.1186/s12916-022-02241-3)
Supplement: Supplementary file 1 — Additional file 1. Tables and text in PDF format. [file 12916_2022_2241_MOESM1_ESM.pdf]

Additional file 1: Supporting Information

Individual, agent-based model

An individual-based model capturing the demographics and movement patterns of individuals within an Australian city, together with SARS-CoV-2 virus transmission data from the early outbreak in China[6], and later with Delta variant specifics[21], was developed and applied. This was used to analyse the effectiveness of a broad suite of pharmaceutical and non-pharmaceutical social distancing interventions (NPIs), by varying age specific vaccination targets in combination with NPIs of varying strength. This model has previously been used to inform policy, both for vaccination and NPIs with influenza[4,16] and NPIs with COVID-19[8]. The modelling method, capturing age, household, school cohort, workplace and community interactions allows for key social distancing measures to be readily represented: school closure; workplace attendance reduction; community contact reduction and case isolation.

This study used a model of Newcastle, a city in New South Wales, Australia (population 273,407), whose population demographics reflect Australia as a whole[3,4] and results were scaled to per million to allow for easy comparison to major population centres. Australian Bureau of Statistics (ABS) census data were used to capture age-specific demographics of every household in the community.[9,10] ABS workplace data was used to assign adults to workplaces, and State Government schools data was used to assign children to age-specific classes.[12] These data were used to model the time-changing contact patterns for each individual, as they move between their household, school/workplace contact hub and in the wider community.

Model parameter settings were calibrated to reflect the transmission characteristics of the COVID-19 pandemic: an incubation period averaging 5 days,[21] from infection to symptom

emergence (if any); a latent period averaging 3 days,[21] from infection to infectious; an infectious period averaging 6 days,[21] the first 2 days being asymptomatic; and 35% of cases are asymptomatic.[22] The probability of virus transmission from infectious to susceptible individuals was derived from an R_0 of 6, based on the relative transmissibility of the Delta variant[2] in comparison to SARS-CoV-2 transmission characteristics from Wuhan, China prior to introduction of containment measures,[6] following the method applied in Milne, Kelso [3].

Model outputs obtained by running the simulation software produced the infection history of every individual in the community, generated the raw daily (and total) number of infectious individuals, where and when infection occurs.[16] Raw infections were translated to detected cases using a ratio of 2:1[13] in order to apply hospitalisation and fatality analyses. Case-hospitalisation and case-fatality ratios for each model age group were calculated as the mean of age-adjusted ratios from UK data over the period of 5th June 2021 to 27th August 2021.[14,15] Derived age-specific case/hospitalisation and case/fatality date are presented in Table S1. Due to the lack of case data in the UK for ages 0 and 1 these ratios were unable to be determined for those age groups, however total hospitalisations and fatalities are understood to be negligible in this age group in comparison to overall population hospitalisations and fatalities. Modelling analyses were then conducted for various combinations of vaccination coverage and the strength of NPIs quantifying the effect of alternative mitigation strategies may have in reducing the impact of the outbreak.

Table S1. Age specific Case/Hospitalisation and Case/Fatality ratios as derived from UK age specific case, hospitalisation and fatality data.[15,16]

Age group	Case / Hospitalisation Ratio	Case / Fatality Ratio
**0 – 6 months**	0.0×10^{-4}	0.0×10^{-6}
**7 – 24 months**	0.0×10^{-4}	0.0×10^{-6}
**3 – 5 years**	1.956×10^{-3}	4.240×10^{-6}
**6 – 12 years**	9.110×10^{-4}	3.704×10^{-6}

**13 – 17 years**	1.501×10^{-3}	2.684×10^{-5}
**18 – 24 years**	1.594×10^{-3}	3.976×10^{-5}
**25 – 44 years**	6.415×10^{-3}	9.577×10^{-5}
**45 – 64 years**	9.704×10^{-3}	9.660×10^{-4}
**65 – 79 years**	2.031×10^{-2}	4.356×10^{-3}
**80+ years**	5.192×10^{-2}	1.590×10^{-2}

Our model depends on certain stochastic parameters, including the probability of virus transmission between an infectious individual and a susceptible individual, and the random seeding of infectious individuals into the community to initiate an outbreak. This results in variation between successive simulation runs. In order to illustrate this variation 100 simulation runs are performed, with Tables S4, S5 and S6 showing the median, 10th and 90th percentile.

Table S2. Cases per million for increasing vaccination coverage levels (ages 12 and up, and 5 and up), showing percentage reductions from a baseline of 70% of 12+. Vaccine with 88% efficacy assumed for all ages. R_0 of=6.0. Median value of 100 simulations presented.

**Cases**		**Total**	**0-12y**	**13-24y**	**25-44y**	**45-64y**	**65-79y**	**80y+**
Total	70% 12+	212,676	67,404	29,831	46,467	43,853	17,480	7,589
	70% 5+	184,781	42,533	29,487	45,412	42,966	17,026	7,372
	80% 12+	164,123	63,021	21,026	33,165	30,391	11,514	4,985
	80% 5+	130,063	34,751	20,252	31,058	28,707	10,694	4,583
	85% 12+	140,219	61,478	16,469	26,302	23,613	8,650	3,718
	85% 5+	99,384	29,362	15,082	23,158	21,083	7,544	3,205
	90% 12+	114,306	57,750	11,773	19,413	16,791	5,962	2,547
	90% 5+	65,119	21,972	9,401	14,585	12,881	4,381	1,869
	95% 12+	113,977	57,524	11,752	19,377	16,805	5,972	2,545
	95% 5+	65,012	21,807	9,473	14,558	12,919	4,423	1,860
Percentage Reduction	70% 12+	*Baseline*						
	70% 5+	13%	37%	1%	2%	2%	3%	3%
	80% 12+	23%	7%	30%	29%	31%	34%	34%
	80% 5+	39%	48%	32%	33%	35%	39%	40%
	85% 12+	34%	9%	45%	43%	46%	51%	51%
	85% 5+	53%	56%	49%	50%	52%	57%	58%
	90% 12+	46%	14%	61%	58%	62%	66%	66%
	90% 5+	69%	67%	68%	69%	71%	75%	75%
	95% 12+	46%	15%	61%	58%	62%	66%	66%
	95% 5+	69%	68%	68%	69%	71%	75%	75%

Table S3. Hospitalisations per million for increasing vaccination coverage levels (ages 12 and up, and 5 and up), showing percentage reductions from a baseline of 70% of 12+. Vaccine with 88% efficacy assumed for all ages. R_0 of=6.0. Median value of 100 simulations presented.

Hospitalisations		Total	0-12y	13-24y	25-44y	45-64y	65-79y	80y+
Total	70% 12+	1,585	65	46	298	426	355	394
	70% 5+	1,522	39	46	291	417	346	383
	80% 12+	1,095	62	33	213	295	234	259
	80% 5+	996	32	31	199	279	217	238
	85% 12+	853	60	26	169	229	176	193
	85% 5+	723	27	23	149	205	153	166
	90% 12+	616	57	18	125	163	121	132
	90% 5+	440	20	15	94	125	89	97
	95% 12+	616	57	18	124	163	121	132
	95% 5+	440	20	15	93	125	90	97
Percentage Reduction	70% 12+	*Baseline*						
	70% 5+	4%	40%	0%	2%	2%	3%	3%
	80% 12+	31%	5%	28%	29%	31%	34%	34%
	80% 5+	37%	51%	33%	33%	35%	39%	40%
	85% 12+	46%	8%	43%	43%	46%	50%	51%
	85% 5+	54%	58%	50%	50%	52%	57%	58%
	90% 12+	61%	12%	61%	58%	62%	66%	66%
	90% 5+	72%	69%	67%	68%	71%	75%	75%
	95% 12+	61%	12%	61%	58%	62%	66%	66%
	95% 5+	72%	69%	67%	69%	71%	75%	75%

Table S4. Deaths per million for increasing vaccination coverage levels (ages 12 and up, and 5 and up), showing percentage reductions from a baseline of 70% of 12+. Vaccine with 88% efficacy assumed for all ages. R_0 of=6.0. Median value of 100 simulations presented.

**Deaths**		**Total**	**0-12y**	**13-24y**	**25-44y**	**45-64y**	**65-79y**	**80y+**
Total	70% 12+	245	0	1	4	42	76	121
	70% 5+	238	0	1	4	42	74	117
	80% 12+	163	0	1	3	29	50	79
	80% 5+	151	0	1	3	28	47	73
	85% 12+	123	0	1	3	23	38	59
	85% 5+	107	0	1	2	20	33	51
	90% 12+	85	0	0	2	16	26	40
	90% 5+	63	0	0	1	12	19	30
	95% 12+	85	0	0	2	16	26	40
	95% 5+	63	0	0	1	12	19	30
Reduction from baseline	70% 12+	*Baseline*						
	70% 5+	3%	-	0%	0%	0%	3%	3%
	80% 12+	33%	-	0%	25%	31%	34%	35%
	80% 5+	38%	-	0%	25%	33%	38%	40%
	85% 12+	50%	-	0%	25%	45%	50%	51%
	85% 5+	56%	-	0%	50%	52%	57%	58%
	90% 12+	65%	-	100%	50%	62%	66%	67%
	90% 5+	74%	-	100%	75%	71%	75%	75%
	95% 12+	65%	-	100%	50%	62%	66%	67%
	95% 5+	74%	-	100%	75%	71%	75%	75%

Table S5. Cases per million for increasing vaccination coverage levels (ages 12 and up, and 5 and up), with and without moderate NPIs. Vaccine with 88% efficacy assumed for all ages. R_0 of=6.0. Median value of 100 simulations presented with 10th and 90th percentiles in brackets.

Cases		Total	0-12y	13-24y	25-44y	45-64y	65-79y	80y+
No NPIs	70% 12+	212,676<br>[212,002-213,398]	67,404<br>[67,124-67,659]	29,831<br>[29,551-30,140]	46,467<br>[46,188-46,849]	43,853<br>[43,588-44,144]	17,480<br>[17,283-17,660]	7,589<br>[7,469-7,711]
	70% 5+	184,781<br>[183,947-185,566]	42,533<br>[42,133-42,900]	29,487<br>[29,131-29,833]	45,412<br>[45,134-45,741]	42,966<br>[42,630-43,234]	17,026<br>[16,807-17,171]	7,372<br>[7,206-7,484]
	80% 12+	164,123<br>[163,323-165,035]	63,021<br>[62,677-63,390]	21,026<br>[20,731-21,348]	33,165<br>[32,841-33,506]	30,391<br>[30,045-30,740]	11,514<br>[11,293-11,688]	4,985<br>[4,879-5,085]
	80% 5+	130,063<br>[128,984-130,963]	34,751<br>[34,221-35,177]	20,252<br>[19,924-20,570]	31,058<br>[30,752-31,369]	28,707<br>[28,424-28,986]	10,694<br>[10,535-10,865]	4,583<br>[4,456-4,711]
	90% 12+	114,306<br>[113,450-115,260]	57,750<br>[57,311-58,274]	11,773<br>[11,484-12,058]	19,413<br>[19,118-19,736]	16,791<br>[16,486-17,107]	5,962<br>[5,852-6,124]	2,547<br>[2,449-2,642]
	90% 5+	65,119<br>[63,156-66,686]	21,972<br>[21,252-22,559]	9,401<br>[9,009-9,752]	14,585<br>[14,132-14,958]	12,881<br>[12,379-13,275]	4,381<br>[4,185-4,580]	1,869<br>[1,748-1,962]
Moderate NPIs	70% 12+	167,682<br>[166,817-168,442]	58,849<br>[58,418-59,254]	24,250<br>[23,872-24,669]	37,429<br>[37,164-37,789]	32,826<br>[32,525-33,280]	10,116<br>[9,937-10,338]	4,107<br>[3,994-4,211]
	70% 5+	129,998<br>[128,663-131,282]	31,373<br>[30,797-31,904]	22,789<br>[22,265-23,244]	33,274<br>[32,903-33,765]	29,880<br>[29,495-30,237]	8,992<br>[8,813-9,204]	3,632<br>[3,529-3,732]
	80% 12+	112,083<br>[110,843-113,182]	49,915<br>[49,316-50,468]	13,918<br>[13,570-14,277]	22,350<br>[22,005-22,763]	18,474<br>[18,110-18,830]	5,233<br>[5,077-5,357]	2,114<br>[2,014-2,195]
	80% 5+	58,322<br>[55,523-60,108]	16,791<br>[15,943-17,519]	9,973<br>[9,389-10,447]	14,465<br>[13,740-15,017]	12,422<br>[11,754-12,873]	3,356<br>[3,159-3,571]	1,319<br>[1,247-1,391]
	90% 12+	64,646<br>[63,368-65,652]	39,580<br>[38,761-40,264]	5,361<br>[5,038-5,599]	9,813<br>[9,563-10,082]	7,200<br>[6,938-7,395]	1,906<br>[1,826-1,996]	759<br>[706-815]
	90% 5+	158<br>[39-471]	50<br>[4-187]	18<br>[2-68]	35<br>[7-98]	28<br>[6-78]	8<br>[2-22]	2<br>[0-9]

Table S6. Hospitalisations per million for increasing vaccination coverage levels (ages 12 and up, and 5 and up), with and without moderate NPIs. Vaccine with 88% efficacy assumed for all ages. R_0 of=6.0. Median value of 100 simulations presented with 10th and 90th percentiles in brackets.

Hospitalisations		Total	0-12y	13-24y	25-44y	45-64y	65-79y	80y+
No NPIs	70% 12+	1,585 [1,569-1,601]	65 [65-66]	46 [46-47]	298 [296-301]	426 [423-428]	355 [351-359]	394 [388-400]
	70% 5+	1,522 [1,503-1,536]	39 [39-39]	46 [45-46]	291 [290-293]	417 [414-420]	346 [341-349]	383 [374-389]
	80% 12+	1,095 [1,078-1,110]	62 [61-62]	33 [32-33]	213 [211-215]	295 [292-298]	234 [229-237]	259 [253-264]
	80% 5+	996 [981-1,012]	32 [31-32]	31 [31-32]	199 [197-201]	279 [276-281]	217 [214-221]	238 [231-245]
	90% 12+	616 [603-631]	57 [57-58]	18 [18-19]	125 [123-127]	163 [160-166]	121 [119-124]	132 [127-137]
	90% 5+	440 [420-456]	20 [20-21]	15 [14-15]	94 [91-96]	125 [120-129]	89 [85-93]	97 [91-102]
Moderate NPIs	70% 12+	1,073 [1,058-1,091]	58 [58-59]	38 [37-38]	240 [238-242]	319 [316-323]	206 [202-210]	213 [207-219]
	70% 5+	940 [923-957]	30 [29-30]	35 [35-36]	213 [211-217]	290 [286-293]	183 [179-187]	189 [183-194]
	80% 12+	611 [596-625]	50 [50-51]	22 [21-22]	143 [141-146]	179 [176-183]	106 [103-109]	110 [105-114]
	80% 5+	382 [361-399]	17 [16-17]	15 [15-16]	93 [88-96]	121 [114-125]	68 [64-73]	68 [65-72]
	90% 12+	260 [251-270]	41 [40-42]	8 [8-9]	63 [61-65]	70 [67-72]	39 [37-41]	39 [37-42]
	90% 5+	1 [0-3]	0 [0-0]	0 [0-0]	0 [0-1]	0 [0-1]	0 [0-0]	0 [0-0]

Table S7. Deaths per million for increasing vaccination coverage levels (ages 12 and up, and 5 and up), with and without moderate NPIs. Vaccine with 88% efficacy assumed for all ages. R_0 of=6.0. Median value of 100 simulations presented with 10th and 90th percentils in brackets.

**Deaths**		**Total**	**0-12y**	**13-24y**	**25-44y**	**45-64y**	**65-79y**	**80y+**
No NPIs	70% 12+	245 [242-248]	0 [0-0]	1 [1-1]	4 [4-4]	42 [42-43]	76 [75-77]	121 [119-123]
	70% 5+	238 [234-241]	0 [0-0]	1 [1-1]	4 [4-4]	42 [41-42]	74 [73-75]	117 [115-119]
	80% 12+	163 [160-166]	0 [0-0]	1 [1-1]	3 [3-3]	29 [29-30]	50 [49-51]	79 [78-81]
	80% 5+	151 [148-154]	0 [0-0]	1 [1-1]	3 [3-3]	28 [27-28]	47 [46-47]	73 [71-75]
	90% 12+	85 [83-88]	0 [0-0]	0 [0-0]	2 [2-2]	16 [16-17]	26 [25-27]	40 [39-42]
	90% 5+	63 [60-66]	0 [0-0]	0 [0-0]	1 [1-1]	12 [12-13]	19 [18-20]	30 [28-31]
Moderate NPIs	70% 12+	146 [143-149]	0 [0-0]	1 [1-1]	4 [4-4]	32 [31-32]	44 [43-45]	65 [63-67]
	70% 5+	130 [127-133]	0 [0-0]	1 [1-1]	3 [3-3]	29 [28-29]	39 [38-40]	58 [56-59]
	80% 12+	77 [74-79]	0 [0-0]	0 [0-0]	2 [2-2]	18 [17-18]	23 [22-23]	34 [32-35]
	80% 5+	49 [47-52]	0 [0-0]	0 [0-0]	1 [1-1]	12 [11-12]	15 [14-16]	21 [20-22]
	90% 12+	29 [27-30]	0 [0-0]	0 [0-0]	1 [1-1]	7 [7-7]	8 [8-9]	12 [11-13]
	90% 5+	0 [0-0]	0 [0-0]	0 [0-0]	0 [0-0]	0 [0-0]	0 [0-0]	0 [0-0]

Table S8. Total cases, hospitalisations and deaths for a population of size 5,000,000, e.g. Greater Sydney or Greater Melbourne. Transmission calibrated to R_0 of 6.0. Vaccine efficacy of 88% assumed for all ages. Median value of 100 simulations presented. Colours are linearly distributed according to the value along a minimum (green)/median (yellow)/maximum (red) scale.

Cases	No Lockdown	Moderate NPIs	Strict NPIs
**70% 12+**	1,063,381	838,410	22,003
**70% 5+**	923,907	649,988	3,047
**80% 12+**	820,615	560,416	899
**80% 5+**	650,314	291,609	587
**90% 12+**	571,530	323,230	367
**90% 5+**	325,597	789	239
**Hospitalisations**	**No Lockdown**	**Moderate NPIs**	**Strict NPIs**
**70% 12+**	7,923	5,367	141
**70% 5+**	7,609	4,699	21
**80% 12+**	5,474	3,053	5
**80% 5+**	4,981	1,910	4
**90% 12+**	3,081	1,301	2
**90% 5+**	2,198	4	1
**Deaths**	**No Lockdown**	**Moderate NPIs**	**Strict NPIs**
**70% 12+**	1,224	728	17
**70% 5+**	1,192	649	3
**80% 12+**	814	385	1
**80% 5+**	755	247	1
**90% 12+**	426	143	0
**90% 5+**	315	0	0

Table S9. Total cases, hospitalisations and deaths per 1,000,000 population Transmission calibrated to R_0 of 2.9 (estimated Alpha strain). Vaccine efficacy of 88% assumed for all ages. Median value of 100 simulations presented. Colours are linearly distributed according to the value along a minimum (green)/median (yellow)/maximum (red) scale.

Cases	No Lockdown	Moderate NPIs	Strict NPIs
**70% 12+**	117,549	229	57
**70% 5+**	81,517	114	45
**80% 12+**	43,392	62	39
**80% 5+**	176	48	39
**90% 12+**	122	51	33
**90% 5+**	39	29	24
**Hospitalisations**	**No Lockdown**	**Moderate NPIs**	**Strict NPIs**
**70% 12+**	778	1	0
**70% 5+**	613	1	0
**80% 12+**	243	0	0
**80% 5+**	1	0	0
**90% 12+**	1	0	0
**90% 5+**	0	0	0
**Deaths**	**No Lockdown**	**Moderate NPIs**	**Strict NPIs**
**70% 12+**	109	0	0
**70% 5+**	87	0	0
**80% 12+**	32	0	0
**80% 5+**	0	0	0
**90% 12+**	0	0	0
**90% 5+**	0	0	0

Table S10. Total cases, hospitalisations and deaths per 1,000,000 population Transmission calibrated to R_0 of 4 (Lower bound of Delta estimate). Vaccine efficacy of 88% assumed for all ages. Median value of 100 simulations presented. Colours are linearly distributed according to the value along a minimum (green)/median (yellow)/maximum (red) scale.

Cases	No Lockdown	Moderate NPIs	Strict NPIs
**70% 12+**	177,572	96,947	126
**70% 5+**	145,131	44,797	95
**80% 12+**	118,337	30,733	52
**80% 5+**	76,675	139	49
**90% 12+**	59,854	181	42
**90% 5+**	158	44	33
**Hospitalisations**	**No Lockdown**	**Moderate NPIs**	**Strict NPIs**
**70% 12+**	1,246	559	1
**70% 5+**	1,139	303	1
**80% 12+**	727	142	0
**80% 5+**	555	1	0
**90% 12+**	281	1	0
**90% 5+**	1	0	0
**Deaths**	**No Lockdown**	**Moderate NPIs**	**Strict NPIs**
**70% 12+**	183	69	0
**70% 5+**	170	39	0
**80% 12+**	102	16	0
**80% 5+**	79	0	0
**90% 12+**	35	0	0
**90% 5+**	0	0	0

Table S11. Total cases, hospitalisations and deaths per 1,000,000 population Transmission calibrated to R_0 of 6 (estimated Delta strain). Vaccine efficacy of 88% assumed for all ages. Median value of 100 simulations presented. Colours are linearly distributed according to the value along a minimum (green)/median (yellow)/maximum (red) scale.

Cases	No Lockdown	Moderate NPIs	Strict NPIs
**70% 12+**	212,676	167,682	4,401
**70% 5+**	184,781	129,998	609
**80% 12+**	164,123	112,083	180
**80% 5+**	130,063	58,322	117
**90% 12+**	114,306	64,646	73
**90% 5+**	65,119	158	48
Hospitalisations	No Lockdown	Moderate NPIs	Strict NPIs
**70% 12+**	1,585	1,073	28
**70% 5+**	1,522	940	4
**80% 12+**	1,095	611	1
**80% 5+**	996	382	1
**90% 12+**	616	260	0
**90% 5+**	440	1	0
Deaths	No Lockdown	Moderate NPIs	Strict NPIs
**70% 12+**	245	146	3
**70% 5+**	238	130	1
**80% 12+**	163	77	0
**80% 5+**	151	49	0
**90% 12+**	85	29	0
**90% 5+**	63	0	0
